# C10orf99 contributes to the development of psoriasis by promoting the proliferation of keratinocytes

**DOI:** 10.1038/s41598-018-26996-z

**Published:** 2018-06-05

**Authors:** Caifeng Chen, Na Wu, Qiqi Duan, Huizi Yang, Xin Wang, Peiwen Yang, Mengdi Zhang, Jiankang Liu, Zhi Liu, Yongping Shao, Yan Zheng

**Affiliations:** 1 Department of Dermatology, the Second Affiliated Hospital, School of Medicine, Xi’an Jiaotong University, Xi’an, China; 2 Department of Dermatology, Shaanxi Provincial People’s Hospital, Xi’an, China; 3 Frontier of institute of science and technology and Key Laboratory of Biomedical Information Engineering of Ministry of Education, School of Life Science and Technology, Xi’an Jiaotong University, Xi’an, China; 4 Department of Dermatology, University of North Carolina, Chapel Hill, NC USA

## Abstract

Psoriasis is a chronic, relapsing inflammatory skin disease. The pathogenesis of psoriasis is complex and has not been fully understood. C10orf99 was a recently identified human antimicrobial peptide whose mRNA expression is elevated in psoriatic human skin samples. In this study, we investigated the functional roles of C10orf99 in epidermal proliferation under inflammatory condition. We showed that C10orf99 protein was significantly up-regulated in psoriatic skin samples from patients and the ortholog gene expression levels were up-regulated in imiquimod (IMQ)-induced psoriasis-like skin lesions in mice. Using M5-stimulated HaCaT cell line model of inflammation and a combinational approach of knockdown and overexpression of C10orf99, we demonstrated that C10orf99 could promote keratinocyte proliferation by facilitating the G1/S transition, and the pro-proliferation effect of C10orf99 was associated with the activation of the ERK1/2 and NF-κB but not the AKT pathways. Local depletion of C10orf99 by lentiviral vectors expressing C10orf99 shRNA effectively ameliorated IMQ-induced dermatitis. Taken together, these results indicate that C10orf99 plays a contributive role in psoriasis pathogenesis and may serve as a new target for psoriasis treatment.

## Introduction

Psoriasis is a chronic, relapsing inflammatory skin disease that affects approximately 2–3% of the world population^1,2^. Psoriasis is characterized by raised, sharply demarcated, erythematous plaques covered with white silvery scale. It is a lifelong disorder associated with multiple comorbidities and considerable psychosocial disability that severely impair the quality of patients’ life^3,4^. Typical histological features of psoriatic skin include hyperkeratosis, parakeratosis, epidermal hyperproliferation, dilation of dermal capillaries and infiltration of inflammatory cells in both dermis and epidermis^5–7^. Although the pathogenesis of psoriasis is complex and has not been fully elucidated, accumulating evidence show that antimicrobial peptides (AMPs), such as LL37, S100 proteins and β-defensins, play important roles in the pathogenesis of psoriasis^8–11^.

C10orf99 (chromosome 10 open reading frame 99), also known as AP-57 (antimicrobial peptide with 57 amino acid residues), was recently identified as a novel human antimicrobial peptide^12^. However, the cellular function of C10orf99 remains largely unknown. One study reported that C10orf99 inhibits colon cancer cell growth^13^. Transcriptomic studies and genomic-scale analysis showed that C10orf99 mRNA is significantly elevated in psoriasis patients, and 2610528A11Rik, the mouse homolog of C10orf99, is also significantly up-regulated in psoriatic mice^14–18^. However, whether C10orf99 is directly involved in the pathogenesis of psoriasis has not been investigated.

In this study, our data showed that C10orf99 was significantly up-regulated in psoriatic skin samples from patients and in IMQ-induced psoriasis-like mice. C10orf99 knockdown in HaCaT cells decreased keratinocyte proliferation by inducing cell cycle arrest under psoriatic inflammation. Overexpression of C10orf99 promoted the proliferation of HaCaT cells by activating two pro-proliferative pathways: the extracellular signal-regulated kinase1/2 (ERK1/2) and NF-κB pathways. Blocking C10orf99 expression ameliorated epidermal hyperplasia, microangiogenesis and the infiltration of inflammatory cells in IMQ-induced psoriasis-like mice. Our results suggested that C10orf99 plays a contributive role in the pathogenesis of psoriasis and may serve as a potential therapeutic target for psoriasis.

## Results

### Expression of C10orf99 is elevated in psoriatic lesions

**Figure 1:**
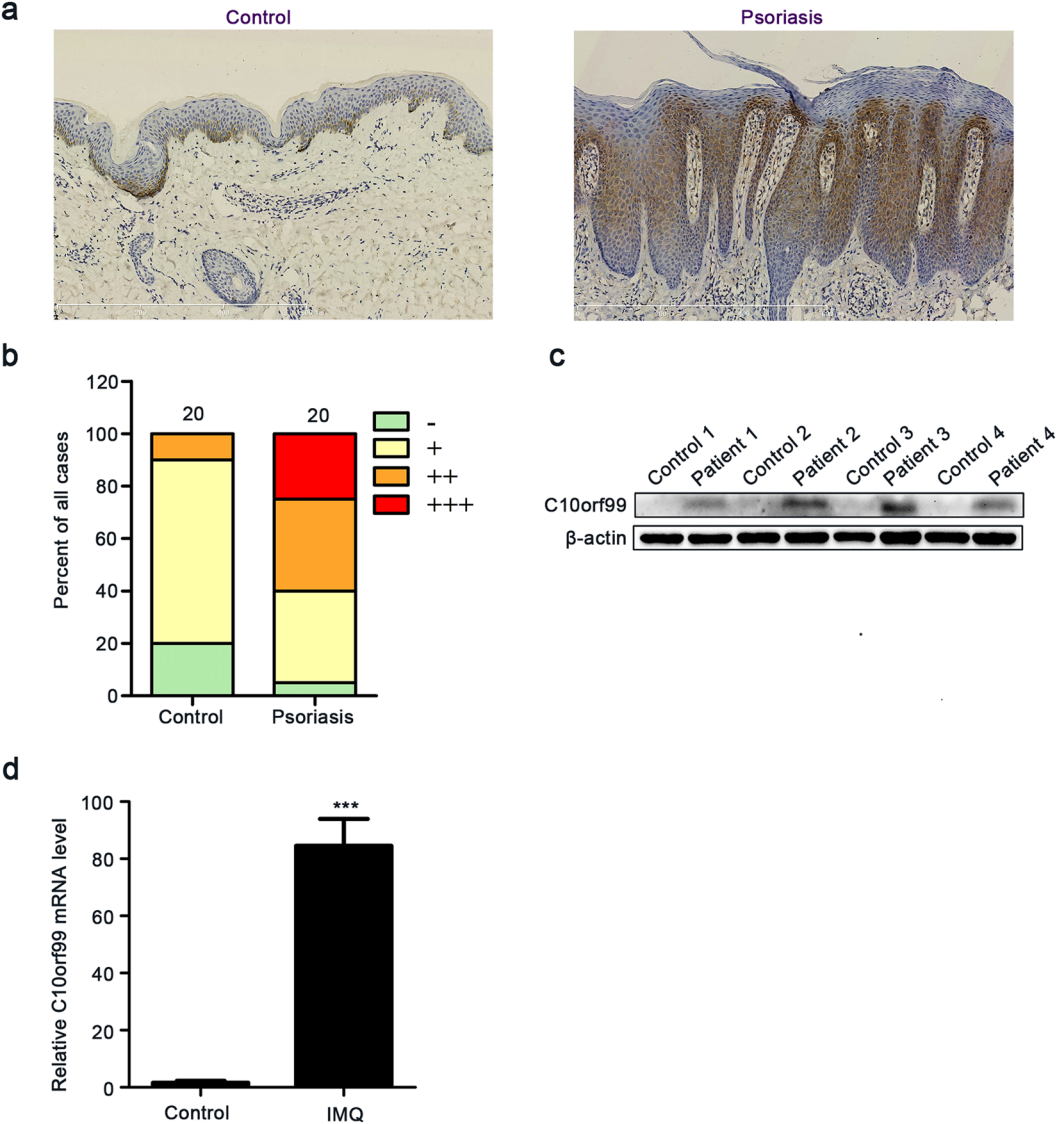
C10orf99 expression in psoriatic lesions. (**a**) Representative immunohistochemistry staining of healthy human skin (n = 20) and psoriatic human skin (n = 20). Bar length = 100 μm. (**b**) Semiquantitative analysis of C10orf99 staining results from 20 healthy and 20 psoriatic skin samples. (**c**) C10orf99 expression was detected by Western blot in skin tissues from psoriasis patients (n = 4) and healthy controls (n = 4). (**d**) C10orf99 expression was evaluated using qRT-PCR in back skins of control (n = 6) and IMQ-induced psoriasis mice (n = 6). ***P < 0.001.

We first examined the expression of C10orf99 at protein level in skin samples obtained from psoriasis patients (n = 20) and healthy donors (n = 20) using immunohistochemical analysis. Staining of C10orf99 was mostly observed in cytoplasm (Fig. 1a). C10orf99 was mainly expressed in the basal layer of the epidermis in the normal skin; however, in psoriasis skin, C10orf99 was over-expressed throughout the thickened epidermis. Semi-quantitative analysis of the immunohistochemistry results indicated that the expression of C10orf99 is remarkably elevated in psoriatic skins compared to the normal controls (Table 1 and Fig. 1b). This observation was further confirmed by comparative western blot analysis of skin samples from 4 psoriasis patients and 4 healthy donors (Fig. 1c). In addition, we analyzed the expression of the mouse homolog of C10orf99, termed 2610528A11Rik, in the IMQ-induced mouse model^19^. Consistently, the expression of 2610528A11Rik was also significantly increased at the mRNA level in the skin lesions from IMQ-treated mice (Fig. 1d). Taken together, these data demonstrate an overexpression of C10orf99 in the psoriatic skin lesions.

**Table 1:** Expression of C10orf99 in Normal skin and Psoriasis.

Group	Case	Expression				*p*-value
		negative	weakly positive	moderately positive	strong positive	
Normal skin	20	4	14	2	0	0.001**
Psoriasis skin	20	1	7	7	5	

Score 0, negative; score 1–4, weakly positive; score 5–8, moderately positive; score 9–12, strong positive.

**P < 0.01, compared to normal skin.

### C10orf99 knockdown inhibits HaCaT cell proliferation by inducing G1/S-growth arrest

**Figure 2:**
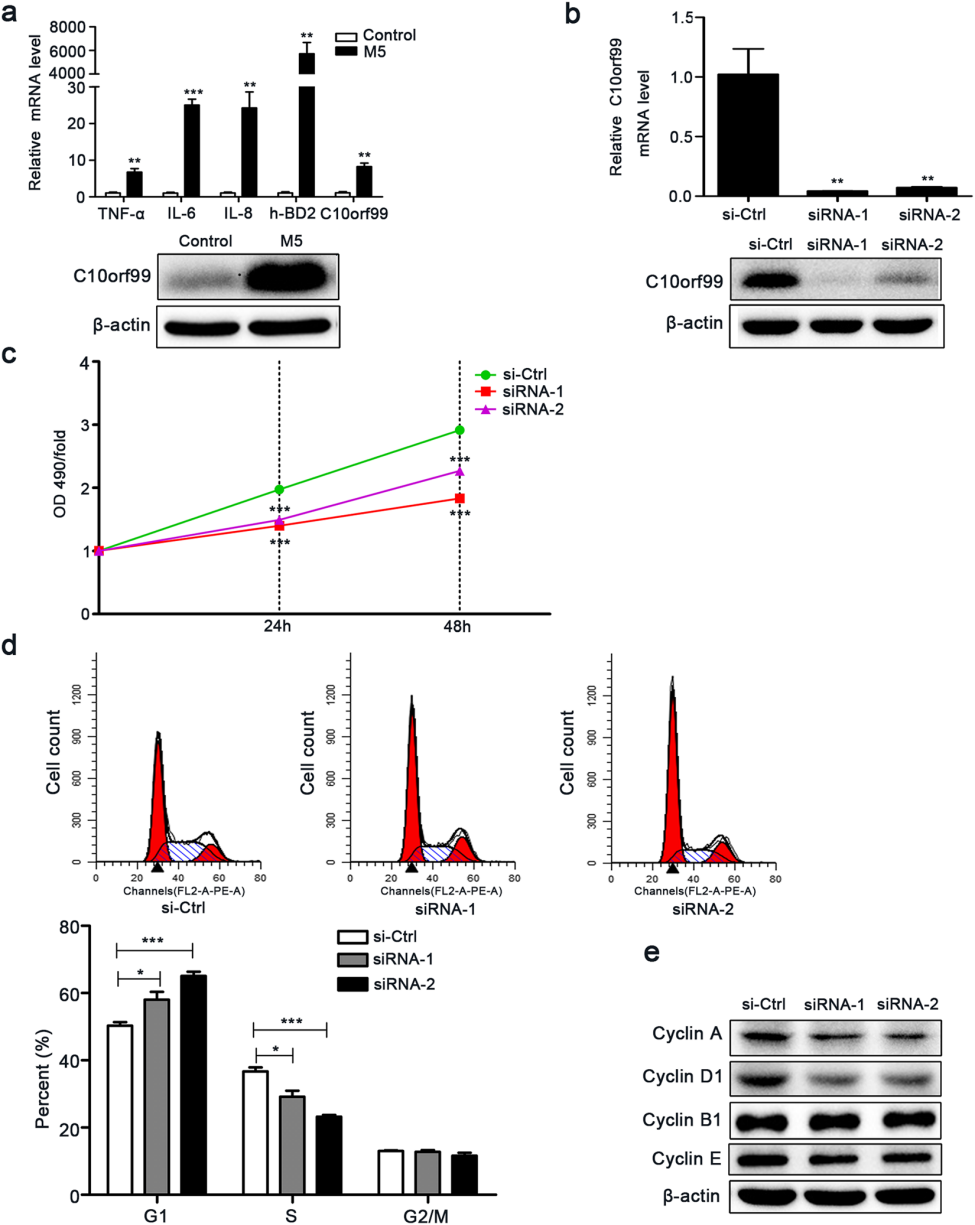
Effect of C10orf99 knockdown on cell proliferation under psoriatic inflammation. (**a**) Expression of inflammatory cytokines and C10orf99 in M5-stimulated HaCaT cells was detected by qRT-PCR (24 h after treatment) and western blot (48 h after treatment). (**b**) C10orf99 mRNA (48 h after transfection) and C10orf99 protein expression (72 h after transfection) was measured in M5-treated HaCaT cells transfected with the C10orf99 small interfering RNA (siRNA) or NC-siRNA (si-Ctrl). (**c**) MTT assays on M5-stimulated HaCaT cells transfected with si-Ctrl or C10orf99 siRNA. (**d**) Cell cycle analysis was analyzed by flow cytometry 72 h after siRNA transfenction. (**e**) Cell cycle regulators were measured by western blot 72 h after siRNA transfenction with β-actin being an interal control. The date are representative of at least three independent experiments. *P < 0.05, **P < 0.01, ***P < 0.001. h, hours; Ctrl, control; OD, optical density; MTT, 3-(4,5-dimethylthiazol-2-yl)−2,5-diphenyltetrazolium bromide.

Enhanced keratinocyte proliferation is an important feature of psoriasis. Since C10orf99 regulates cell proliferation in cancer cells^13^ and is significantly overexpressed in psoriatic skins, we determined whether C10orf99 may contribute to the development of psoriasis by regulating keratinocyte proliferation. We first tested its role in a well-established cell culture model of inflammation that recapitulates some features of psoriasis by stimulating HaCaT cells with a cocktail of cytokines M5 (including TNF-α, IL-17A, IL-22, IL-1α, and Oncostatin-M, 10 ng/ml)^20,21^. As shown previously, expressions of TNF-α, IL-6, IL-8 and h-BD2 were markedly increased in HaCaT cells after 24 h treatment of M5 (Fig. 2a)^20^. More importantly, both western blot and qRT–PCR analysis showed that C10orf99 expression was greatly elevated in M5-stimulated HaCaT cells (Fig. 2a). Thus, HaCaT cells stimulated with M5 could serve as a cell culture model to investigate the role of C10orf99 in the proliferation of keratinocytes.

We then used two independent C10orf99-specific siRNAs to transiently decrease C10orf99 expression in M5-stimulated HaCaT cells. Western blot and qRT-PCR results confirmed the effective down-regulation of C10orf99 by both siRNAs (Fig. 2b). In the absence of the inflammatory cytokine cocktail M5, depletion of C10orf99 had none (siRNA-1) or very weak inhibitory effect (siRNA-2) on HaCaT cell growth (Supplementary Figure S5), presumably due to a very low basal expression level of C10orf99. Previous studies have shown that M5 treatment accelerates the growth of HaCaT cells by around 1.5 fold^22,23^. Notably, C10orf99 knockdown significantly reduced the growth rate of M5-stimulated HaCaT cells as demonstrated by the MTT assays (Fig. 2c). Cell cycle analysis by PI staining further revealed that C10orf99 knockdown led to a G1/S growth arrest accompanied by reduced expressions of G1-S progression regulators such as Cyclin D1 and Cyclin A (Fig. 2d,e). Thus, C10orf99 depletion inhibits the growth of keratinocytes under inflammatory conditions.

### C10orf99 downregulation has no effect on keratinocyte apoptosis

We also assessed the effect of C10orf99 downregulation on keratinocyte apoptosis by Annexin V/PI staining. However, no significant difference in apoptosis rate was observed between C10orf99-depleted and control cells, demonstrating that C10orf99 knockdown did not affect HaCaT cell apoptosis, ruling out a possibility that the reduced growth rate in C10orf99 knockdowned HaCaT cells was caused by increased apoptosis (Supplementary Figure S1).

### Overexpression of C10orf99 promotes the proliferation of HaCaT cells

**Figure 3:**
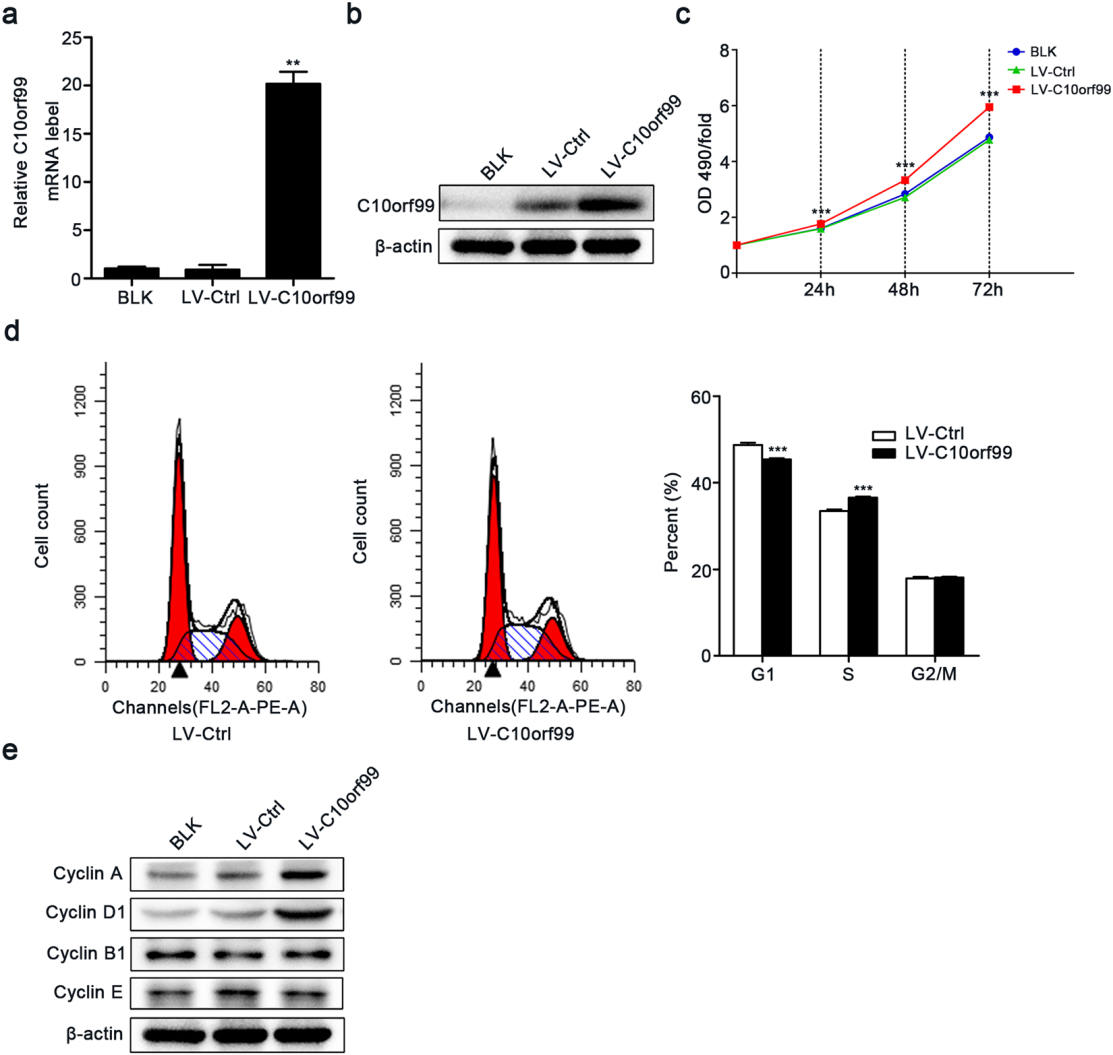
Effect of C10orf99 overexpression on cell proliferation. (**a**) C10orf99 mRNA and (**b**) protein expressions were determined after lentiviral particles transduction in HaCaT cells. (**c**) MTT assays on HaCaT cells transduced with control or C10orf99-expressing lentiviruses. (**d**) Cell cycle distribution was analyzed by flow cytometry, and the percentages of cells in different cycle phases were calculated. (**e**) Protein expression levels of cell cycle regulators were analyzed. Date are representative of at least three independent experiments. **P < 0.01, ***P < 0.001. BLK, blank transfected group; LV-Ctrl, vector transfected group; LV-C10orf99, C10orf99-expressing lentiviruses transfected group.

To further confirm the role of C10orf99 in the regulation of KC proliferation, we over-expressed C10orf99 via a lentiviral vector in HaCaT cells without M5 treatment, which express low basal level of C10orf99. Lentivirus-mediated expression of exogenous C10orf99 was verified by qRT-PCR and western blot analysis (Fig. 3a,b). MTT assay showed that overexpression of C10orf99 increased the proliferation of HaCaT cells as compared with the blank or the empty virus control (Fig. 3c). PI cell cycle analysis revealed that overexpression of C10orf99 resulted in an increased S phase population with a concomitant decrease in the G1/G0 phase population (Fig. 3d). As expected, the expressions of the G1/S progression regulators, Cyclin D1 and Cyclin A were increased in C10orf99 over-expressed HaCaT cells versus the control cells (Fig. 3e). Therefore, C10orf99 overexpression promotes the proliferation of HaCaT cells by facilitating the G1/S progression.

### C10orf99 activates the ERK1/2 and the NF-κB signaling

**Figure 4:**
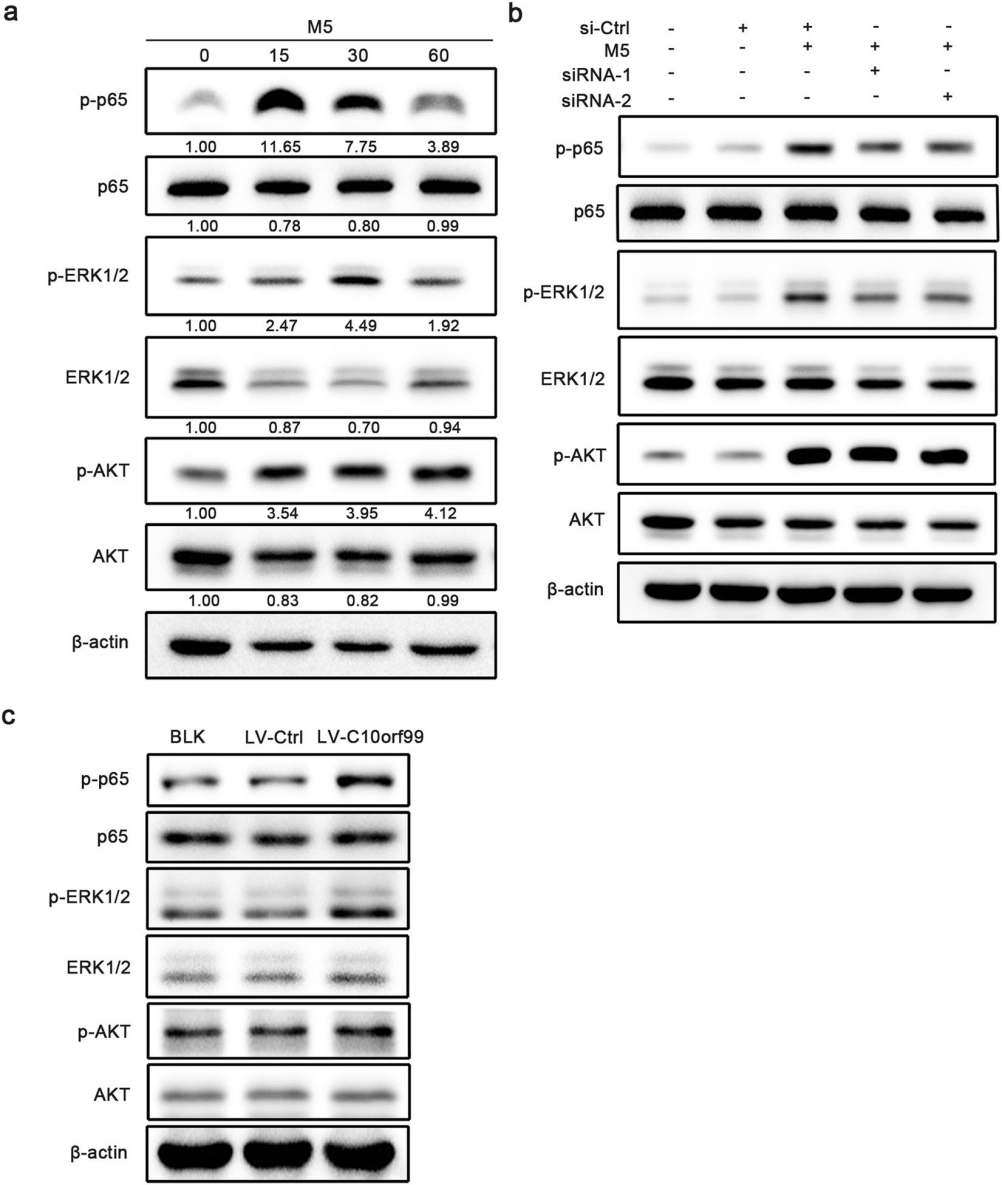
C10orf99 regulates the keratinocyte proliferation by activating ERK1/2 and NF-κB pathways. (**a**) HaCaT cells were treated with M5 for 15, 30 and 60 min, and the phosphorylation of NF-κB p65, ERK1/2 and AKT were detected by western blot. (**b**) HaCaT cells were transfected with C10orf99 siRNAs for 48 h and then incubated with M5 for 30 min. Cells were lysed for western blot analysis on indicated proteins. (**c**) Phosphorylation of NF-κB p65, ERK1/2 and AKT were analysed by western blot in HaCaT cells transduced with lentiviral particles. β-actin was used as an internal control. Date are representative of three independent experiments. Ctrl, control; BLK, blank transfected group; LV-Ctrl, vector transfected group; LV-C10orf99, C10orf99-expressing lentiviruses transfected group.

We initially evaluated phospho-p65, phosphor-ERK1/2 and phosphor-AKT expressions in M5-treated HaCaT cells by western blot. We observed that all these pathways were prominently activated after incubation with M5 for 15 to 30 min (Fig. 4a). To study the molecular mechanisms underlying the pro-proliferation function of C10orf99, we used C10orf99-specific siRNAs to decrease the expression of C10orf99 followed by treated with M5 for 30 min and analyzed several key proteins involved in the signaling pathways. The data demonstrated that knockdown of C10orf99 suppressed the activity of ERK1/2 and NF-κB signaling, as evidenced by the reduction of the levels of p-ERK1/2 and p-p65, but not the AKT pathway (Fig. 4b). Conversely, over-expression of C10orf99 increased the levels of p-ERK1/2 and p-p65 (Fig. 4c). Therefore, the pro-proliferation activity of C10orf99 is associated with the activation of the ERK1/2 and NF-κB pathways.

### C10orf99 knockdown ameliorates imiquimod-induced dermatitis

**Figure 5:**
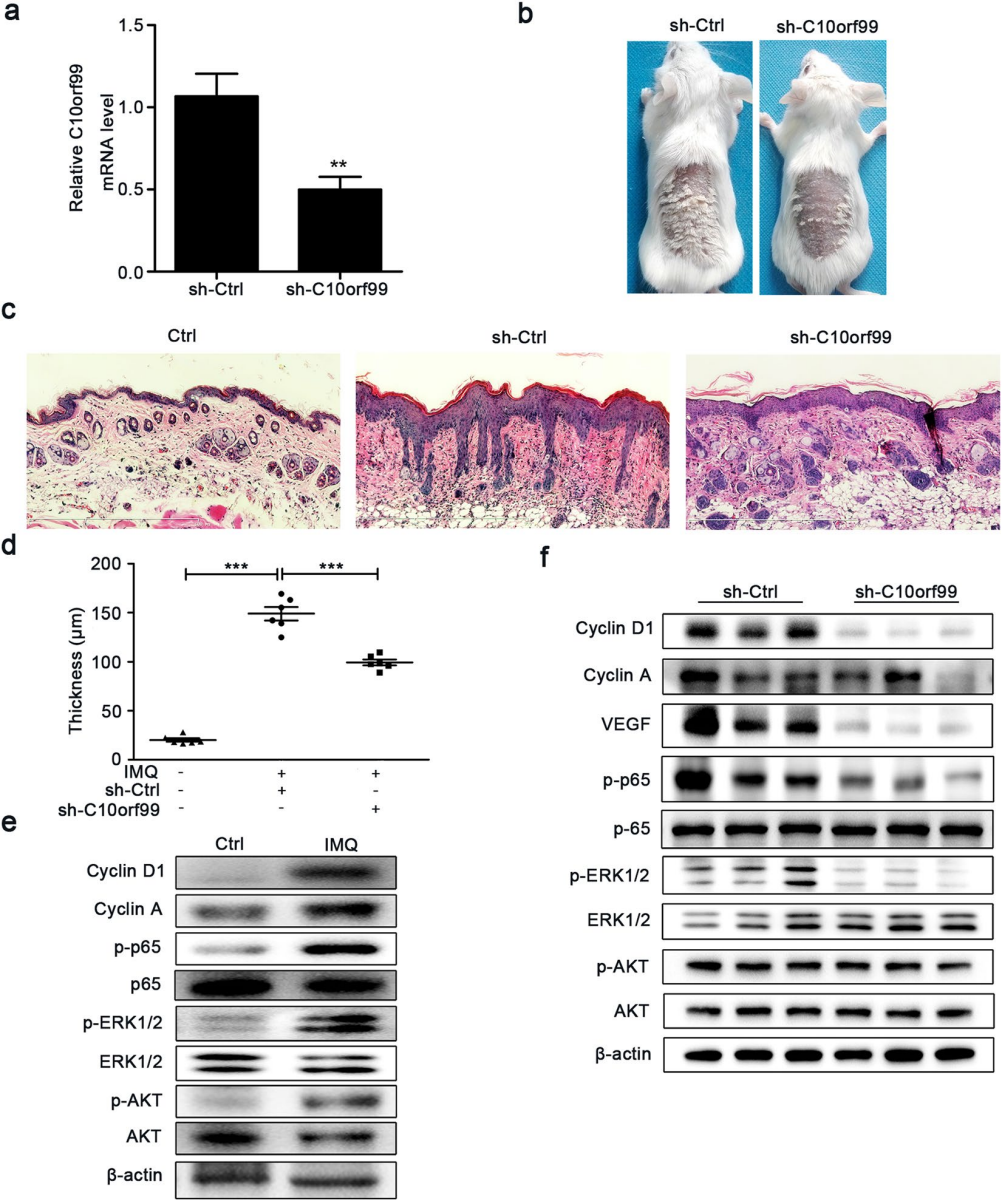
C10orf99 knockdown reduced the epidermal thickness of IMQ-induced psoriasis mouse model. (**a**) qRT-PCR analysis of mC10orf99 expression in the back skins of mice infected with the lentiviruses and treated with IMQ (7 days). (**b**) Phenotypic presentation of the back skins of mice after lentivirus infection and IMQ treatment for 7 days. (**c**) Reprsentative histological sections of the back skins of control (Ctrl) (n = 6) or IMQ-induced psoriatic mice injected intradermally with C10orf99 shRNA (sh-C10orf99) (n = 6) or negative control shRNA (sh-Ctrl) (n = 6) examined by hematoxylin and eosin staining. Bar = 600 μm. (**d**) Epidermal thickness of the mouse back skins was measured. Each point represents one mouse skin sample. (**e**) Western blot analysis of Cyclin A, Cyclin D1, NF-κB p65, phosphorylation of NF-κB p65, AKT, ERK1/2 in skin tissues from control and IMQ-induced psoriatic mice. (**f**) Western blot analysis of Cyclin A, Cyclin D1, VEGF and phosphorylation of NF-κB p65, AKT, ERK1/2 in skin tissues from IMQ-induced psoriatic mice injected intradermally with lentiviruses. β-actin was used as an internal control. Date are representative of three independent experiments. **P < 0.01, ***P < 0.001.

To determine whether C10orf99 participates in the pathogenesis of psoriasis by promoting keratinocyte proliferation, we locally knocked down C10orf99 expression in mouse back skin by injecting lentiviral particles carrying the C10orf99 shRNA and examined the impact of C10orf99 depletion on the development of IMQ-induced psoriasis^24,25^. The knockdown efficiencies of three shRNAs were first verified in a mouse cell line C2C12 which expresses endogenous C10orf99. Out of three shRNAs, shRNA-1 showed the best knockdown efficiency and was therefore chosen for the *in vivo* experiment (Supplementary Figure S2). The *in vivo* knockdown efficiency of shRNA-1 was confirmed by qRT-PCR analysis on skin tissues from the virus-injected area (Fig. 5a). IMQ treatment induced sharply demarcated erythematous plaques covered with white silvery scale on mouse back. However, C10orf99 knockdown led to a significant decrease in plaque formation (Fig. 5b). Histological analysis of the skin sections revealed that the epidermal hyperplasia was ameliorated, the epidermal thickness was decreased and microangiogenesis and the infiltration of inflammatory cells were reduced in C10orf99-depleted mice compared to the control mice (Fig. 5c,d). Prominent increases in p-p65, p-ERK1/2, p-AKT, Cyclin A and Cyclin D1 levels were observed after IMQ applied for 7 consecutive days (Fig. 5e). In addition, we found that C10orf99 knockdown down-regulated the expression of Cyclin D1 and the angiogenesis regulator VEGF (Fig. 5f). Moreover, consistent with the results *in vitro*, knock-down of C10orf99 by shRNA decreased the activity of ERK1/2 and NF-κB signaling, as demonstrated by downregulation of p-ERK1/2 and p-p65 (Fig. 5f). These data further supported that C10orf99 plays an important role in the development of psoriasis.

## Discussion

C10orf99 was recently identified as a new human AMP^12^. Its gene is located on chromosome 10q23.1 and encodes a short basic peptide of 57 amino acid residues^12^. It has been found that C10orf99 mRNA has distinct tissue-specific expression patterns: highly expressed in colon, moderately expressed in tonsil and almost undetectable in other tissues^13^. Like other AMPs, such as LL-37 and h-BD2, C10orf99 also exhibits potent wide-spectrum antimicrobial activity against bacteria, fungi and viruses^12^. A study of large-scale mouse knockout library screen implicated C10orf99 in the immune regulation, for C10orf99 knockout mouse exhibits a decreased serum IgM level and an increased ratio of CD4+/CD8+ cells^26^. Similar to other AMPs, *in situ* gel-forming AP-57 peptide promotes cutaneous wound healing^27–30^. Recently, C10orf99 was speculated to participate in the pathogenesis of psoriasis without the support of any experimental data^15^. Therefore, it is of great interest to investigate the function of C10orf99 in the development of psoriasis.

In this work, we demonstrated that the expressions of C10orf99 and its ortholog were significantly up-regulated in the skin of psoriasis patients and IMQ-induced psoriatic mouse models respectively. We also showed that C10orf99 positively regulates keratinocyte proliferation. Paradoxically, C10orf99 was previously found to be growth-inhibitory in colon cancer cells^13^. The proliferation of keratinocyte is a complex process regulated by a variety of intracellular/extracellular agents including growth factors, neuropeptides, interleukins and inflammatory mediators, many of which may play versatile roles in different cellular/environmental contexts. It is not unusual that a protein performs opposite functions in the proliferation of two different epithelial cell lines. For instance, studies have shown that the S100A8/S100A9 complex, which has a broad range of intracellular and extracellular functions, suppresses keratinocyte proliferation, but promote the growth and metastasis of colorectal cancer cells^31,32^. How can the contradictory role of C10orf99 on cell proliferation be explained? The previous paper claimed that C10orf99 could inhibit colon cancer cell growth via interacting with SUSD2^13^. However, a transcriptome analysis demonstrated that SUSD2 was significantly down-regulated in psoriatic lesions^18^. In line with this, we observed that the expression of SUSD2 mRNA also decreased in M5-treated HaCaT cells (Supplementary Figure S3). Thus, it’s likely that C10orf99 regulates keratinocyte proliferation through a distinct mechanism independent of the SUSD2 pathway. Furthermore, knockdown of C10orf99 in the colon cancer cell line DLD1 does not alter the ERK1/2 pathway activity (Supplementary Figure S4). Interestingly, recently studies found that C10orf99 is a natural ligand for the orphan receptor GPR15^33,34^. We speculated that, like other AMPs and chemokines, C10orf99 may function through different receptor pathways in different cellular contexts and exert versatile impacts on the proliferation of cells.

AKT, ERK1/2 and NF-κB signaling pathways play important roles in many biological functions. Studies have shown that the expression of all these protein kinases were significantly elevated in psoriatic skin and they play critical roles in the pathgenesis of psoriasis^35–39^. Our results *in vitro* showed that M5 greatly activated the AKT, ERK1/2 and NF-κB signaling pathways. C10orf99 signaling was previously speculated to involve the AKT pathway. Interestingly, our data showed that knockdown of C10orf99 decreased the level of p-ERK1/2, but not p-AKT, in the cell culture model of psoriasis. In addition, C10orf99 knockdown resulted in a significant reduction of p-p65, indicative of the down-regulation of the NF-κB pathway activity. Conversely, over-expression of C10orf99 promoted the activity of ERK pathway and NF-κB pathway. Thus, C10orf99 likely regulates the proliferation of keratinocyte by activating the ERK1/2 and the NF-κB signaling.

The regulatory role of C10orf99 in keratinocyte proliferation *in vitro* prompted us to assess its functionality *in vivo*. The IMQ-induced psoriasis-like mouse model recapitulates many features of human psoriasis and has been widely used to study the disease pathology. In the IMQ-treated mice, C10orf99 knockdown attenuated epidermal hyperproliferation, infiltration of inflammatory cells and microangiogenesis, which was accompanied by the decreased expression of VEGF. C10orf99 knockdown also decreased the expression of Cyclin D1, p-ERK1/2 and p-p65. However, the knockdown of C10orf99 had marginal effects on Cyclin A, which likely due to different cellular environments between *in vitro* and *in vivo*.

In summary, our results show that C10orf99 protein is highly expressed in psoriatic skin and that it regulates keratinocyte proliferation likely through activation of the ERK1/2 and NF-κB pathway. Furthermore, downregulation of C10orf99 ameliorates IMQ-induced psoriasis in mice. Thus, C10orf99 has a contributive role in psoriasis pathogenesis and may be a new target for psoriasis treatment.

## Materials and Methods

### Human skin tissue collection

A total of 40 paraffin-embedded tissues including 20 psoriasis tissues (11 male and 9 female, age range = 7–50 years) and 20 normal skin tissues (10 male and 10 female, age range = 10–43 years) for IHC were obtained from the tissue bank of the Department of Dermatology at the Second Affiliated Hospital of Xi’an Jiaotong University. In addition, we collected 8 fresh tissue samples including 4 psoriasis samples and 4 normal samples by punch biopsy under local lidocaine anaesthesia for Western blotting. The fresh tissue samples were snap frozen in liquid nitrogen and stored at −80 °C. Written informed consents were obtained from all patients. The study was performed in accordance with the declaration of Helsinki Principles and approved by the Research Ethics Board of the Second Affiliated Hospital of Xi’an Jiaotong University. All of the specimens were pathologically confirmed.

### Immunohistochemistry

Immunohistochemistry was performed according to standard methods. The IHC results were scored independently by three experienced pathologists under microscope and quantified based on the following scoring system in a semiquantitative manner as previously reported^40,41^. The rate of positively stained cells was scored as follows: 0 (≤5%), 1 (6–25%), 2 (26–50%), 3 (51–75%), 4 (>75%). The staining intensity was graded as follows: 0 (colorless), 1 (light yellow), 2 (yellowish brown), 3 (chocolate brown). The score for each microscopic field was calculated by multiplying the two scores. The average score of five fields was taken as the final immunoreactivity score.

### Histological analysis

The mouse back skin was collected, fixed in formalin and embedded in paraffin. Sections were stained with haematoxylin and eosin. Epidermal hyperplasia (acanthosis) was quantified by measuring its thickness as described previously^42^. Firstly, Hamamatsu digital pathology system was used to scan the HE-stained sections. Then, the NDP.view software was used to evaluated the epidermis thickness and it measured the distance from the basal lamina to the bottom of the stratum corneum in HE-stained skin sections. Eight randomly-chosen fields of each section were measured and the mean was calculated.

### Cell culture

HaCaT cells (an immortalized human keratinocyte cell line), C2C12 cells (mouse muscle cell line) and DLD1 cells were cultured in Dulbecco’s modified Eagle’s medium supplemented with 10% fetal bovine serum and 1% penicillin/streptomycin at 37 °C in a humidified atmosphere with 5% CO2.

### Induction of psoriatic model *in vitro*

HaCaT cells were stimulated with a cocktail of cytokines, M5, which includes TNF-α, IL-17A, IL-22, IL-1α, and Oncostatin-M (Peprotech, USA) to induce psoriatic inflammation^20,21^.

### siRNA transfection

The siRNAs for C10orf99 and a nonsilencing-siRNA used as the negative control were synthesized by GenePharma (Shanghai,China) and the sequences were listed in Supplementary Table S1. The siRNAs were transiently transfected into cells using Lipofectamine RNAiMAX (Invitrogen) according to the manufacturer’s instructions. After 24 h for transfection, M5 was added to stimulate the transfected cells.

### Lentivirus transduction

The C10orf99-overexpressing lentivirus particles were purchased from GenePharma (Shanghai, China). Cells were incubated with 100ul of the virus suspension (titer 1 × 10^8^ TU/ml) for 48 h and selected with puromycin (5 mg/ml) in the culture medium for 2 weeks.

### 3-(4,5-dimethylthiazol-2-yl)-2,5-diphenyltetrazolium bromide (MTT) assay

4000 transfected cells per well were plated in 96-well plate, which were then incubated at 37 °C, 5% CO2. At the indicated time points, 10% volume of MTT solution (5 mg/ml) was added to each well and incubated at 37 °C for 4 h. Then 200 μL dimethlsulfoxide was added to dissolve the formazan crystals after removing the medium. Optical density (OD) value was measured at 490 nm wavelength.

### PI cell cycle analysis

Cells were harvested and fixed in cold 70% ethanol at −20 °C overnight. For the analysis, PI staining solution (50 mg/mL PI and 100 mg/mL ribonuclease A) was added to the cells and incubated at 37 °C for 30 minutes in dark. Cell cycle was analyzed using flow cytometry (FACSCalibur from BD Biosciences, USA).

### Annexin V/PI staining

Cells were harvested by trypsinization without ethylenediaminetetraacetic acid (EDTA). The cells then were suspended by 500 μl binding buffer and incubated with 5 μl Annexin V-FITC (fluorescein isothiocyanate) solution and 5 μl Propidum Iodidum for 15 min at room temperature in dark. Flow cytometry was used to analyze cell apoptosis.

### RNA extraction and Quantitative Real-Time PCR

Total RNA was extracted from skin samples or HaCaT cells using Trizol reagent (Invitrogen). Quantitative realtime PCR (qRT-PCR) was performed on a real-time PCR system (Eppendorf, Germany) using SYBR Green Premix Ex Taq (TaKaRa, Dalian, China). Primers were synthesized by Baiaoke Biotech (Beijing, China) and the following primers were used: human GAPDH: forward 5′-TGTTGCCATCAATGACCCCTT-3′, and reverse 5′-CTCCACGACGTACTCAGCG-3′; human C10orf99: forward 5′-GCTTCTCTGCTTCTCCATCTTCT-3′, and reverse 5′-TTCAGGTTTGTTGAGTTGGG-3′; human TNF-α: forward 5′-TCCTTCAGACACCCTCAACC-3′; and reverse 5′-AGGCCCCAGTTTGAATTCTT-3′; human IL-6: forward 5′-AAGCCAGAGCTGTGCAGATGAGTA-3′, and reverse 5′-TGTCCTGCAGCCACTGGTTC-3′; human IL-8: forward 5′-GTCCTTGTTCCACTGTGCCT-3′, and reverse 5′-GCTTCCACATGTCCTCACAA-3′; human BD2: forward 5′- TCAGCCATGAGGGTCTTGTA-3′, and reverse 5′-GGATCGCCTATACCACCAAA-3′; mouse 2610528A11Rik: forward 5′- TTCTAGCCCTTTCCGGTCTG-3′, and reverse 5′- CACCACCCATGACTTGACTG-3′; mouse β-actin: forward 5′-CCTCTATGCCAACACAGTGC-3′, and reverse 5′- ACATCTGCTGGAAGGTGGAC-3′. GAPDH or β-actin was used as internal controls. The relative quantification of gene was obtained using the 2^−ΔΔCT^ method relative to the internal control.

### Western blot

Total protein was prepared from cells or skin samples using RIPA lysis buffer containing 1 mM PMSF and the protein was quantified by BCA protein assay. The prepared proteins (15 μg) were separated by 10–12% SDS-PAGE and transferred to nitrocellulose membrane or polyvinylidene difluoride membrane. The membrane was blocked with TBST containing 5% skim milk for 1 h at room temperature and incubated with the primary antibodies at 4 °C overnight. The following primary antibodies were used: Cyclin A (sc-596), Cyclin B1 (sc-595), Cyclin D1 (sc-246), Cyclin E (sc-25303), NF-κB p65 (sc-71675), p-NF-κB p65 (#3033), AKT (#9272 s), p-AKT (#4051 s), ERK1/2 (#9102), p-ERK (#3192 s), VEGF (sc-7269), β-actin (60008–1-Ig), C10orf99 (ab151109). The membrane was then incubated in horseradish peroxidase-conjugated goat anti-mouse or anti-rabbit secondary antibodies at room temperature for 1 h. ECL kit (Pierce Chemical, Rockford, IL, USA) was used to perform chemiluminent detection.

### Mice

Eight-week-old female BALB/c mice were purchased from the Animal Experiment Center of Xi’an Jiaotong University and they were kept under a 12 h light/dark cycle with specific pathogen-free conditions. All mice were housed at least 1 week prior to the study and provided with food and purified water ad libitum. All procedures involved in mice were performed in compliance with the Animal Care and Use Committee guidelines of Xi’an Jiaotong University School of Medicine.

### IMQ-induced mouse model of psoriasis

Mice were anesthetizes with 200 ul 0.6% sodium pentobarbital and their backs were shaved with an electric clipper, then the mice were applied with a daily topical dose of 62.5 mg 5% IMQ cream (Mingxin, Chengdu, China) or Vaseline cream on their shaved back for 7 consecutive days. In the end, all mice were sacrificed. Back skin was isolated and half was fixed in 10% formaldehyde and the other half of the skin sample was finely chopped for RNA isolation and qRT-PCR analysis.

### *In vivo* administration of C10orf99 shRNA lentivirus

Lentiviruses of C10orf99 shRNAs and control shRNAs were from GenePharma (Shanghai,China) and all the oligonucleotides were listed in supplementary Table S2. shRNA-1 successfully knocked down the C10orf99 mRNA expression level and was used in animal experiment. Lentivirus particles (1.0 × 109 TU, 50 μL) encoding C10orf99 shRNA or negative control shRNA were injected intradermally into the shaved dorsal skin of mice. After three days, the mice started to be treated with IMQ as described above.

### Statistical analysis

SPSS standard version 21.0 software (SPSS Inc, Chicago, IL) and GraphPad Prism 5.0 (La Jolla, CA, USA) were used for statistical analysis. Data were presented as mean ± SEM. The Mann–Whitney U test was used for immunohistochemistry analysis and the Student’s t-test was used for comparisons between two groups. *P* < 0.05 was considered statistically significant.

### Data availability

All data generated or analysed during this study are included in this published article (and its Supplementary Information files).

## Electronic supplementary material

Supplementary information
