# Supplementary material for: C10orf99 contributes to the development of psoriasis by promoting the proliferation of keratinocytes
Source: Sci Rep. 2018 Jun 5;8:8590. doi: 10.1038/s41598-018-26996-z (PMC5988722; doi:10.1038/s41598-018-26996-z)
Supplement: Supplementary file 1 — Supplementary information [file 41598_2018_26996_MOESM1_ESM.pdf]

C10orf99 contributes to the development of psoriasis by promoting the proliferation of keratinocytes

Caifeng Chen[1], Na Wu[2], Qiqi Duan[1], Huizi Yang[3], Xin Wang[1], Peiwen Yang[1], Mengdi Zhang[1], Jiankang Liu[3], Zhi Liu[4], Yongping Shao[3*], Yan Zheng[1*]

[1]Department of Dermatology, the Second Affiliated Hospital, School of Medicine, Xi'an Jiaotong University, Xi'an, China;

[2]Department of Dermatology, Shaanxi Provincial People's Hospital, Xi'an, China;

[3]Frontier of institute of science and technology and Key Laboratory of Biomedical Information Engineering of Ministry of Education, School of Life Science and Technology, Xi'an Jiaotong University, Xi'an, China;

[4]Department of Dermatology, University of North Carolina, Chapel Hill, NC, USA.

Supplemental Tables

Table S1. The sequences of siRNAs used in the study

Name	Sequences
siRNA-1	Forward: 5′-GACCAUCAAUCCUGCUAGATT-3′
	Reverse: 5′-UCUAGCAGGAUUGAUGGUCTT-3′
siRNA-2	Forward: 5′-GACAUGAGUCUGCUGGAAATT-3′
	Reverse: 5′-UUUCCAGCAGACUCAUGUCTT-3′
Negative Control	Forward: 5′-UUCUCCGAACGUGUCACGUTT-3′
	Reverse: 5′-ACGUGACACGUUCGGAGAATT-3′

Table S2. The sequences of shRNAs used in the study

Name	Sequences
shRNA-1	5’-GGAGAAGACATCCTGCCAAGT-3’
shRNA-2	5’-GCTGTCACCTATCTCCTAGAT-3’
shRNA-3	5’-GCAGAAACAAGCTACCAGTCA-3’
Negative Control	5’-TTCTCCGAACGTGTCACGT-3’

Supplemental Figures

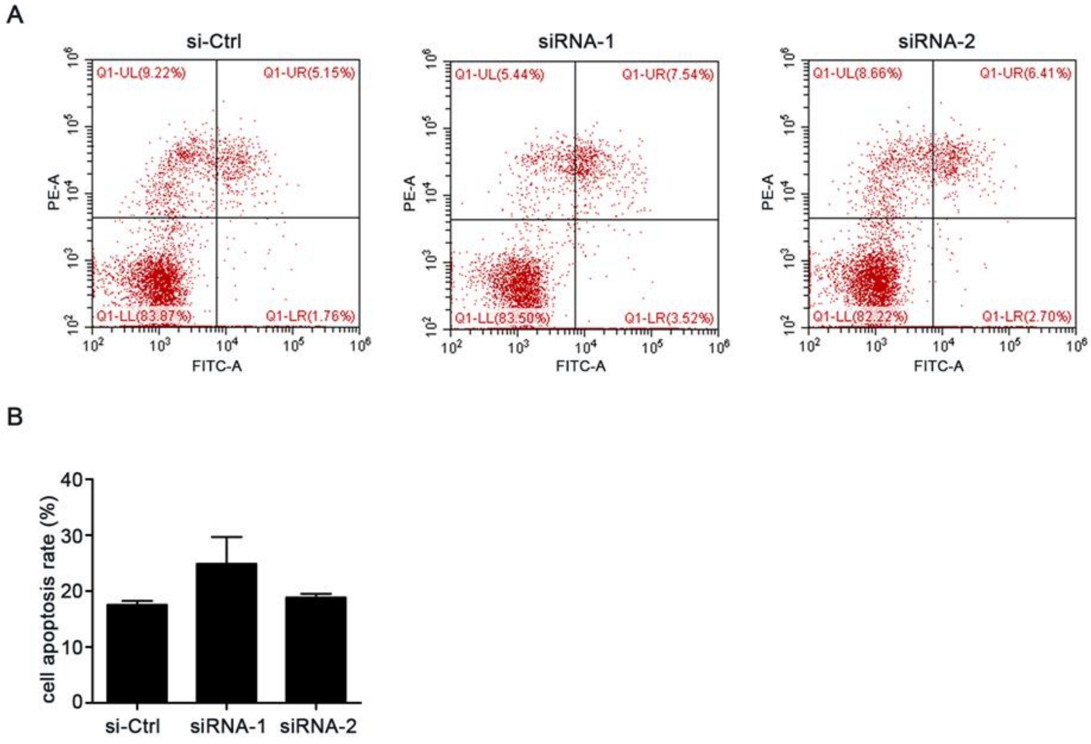

Figure S1. Effect of C10orf99 knockdown on cell apoptosis under psoriatic inflammation. (A) Cell apoptosis analysis of M5-stimulated HaCaT cells transfected with control or C10orf99 siRNAs. **(B)** Quantification of the results from **A**.

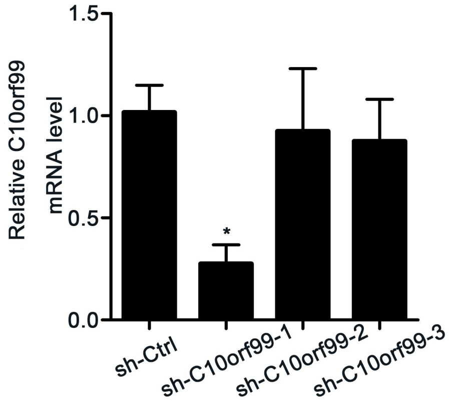

Figure S2. The knockdown efficiencies of C10orf99 lentivirus shRNA. C2C12 cells were transduced with lentiviral particles expressing different shRNAs and the knockdown efficiencies of three shRNAs were verified by qRT-PCR. *P <0.05.

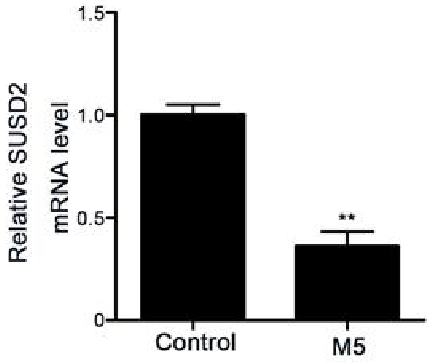

Figure S3. The mRNA expression level of SUSD2 in HaCAT cells treated with or without M5. HaCaT cells were stimulated with or without M5 for 24 h and the expression of SUSD2 mRNA was detected by qRT-PCR. ***P* <0.01.

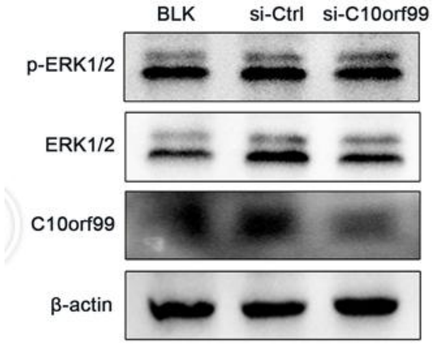

Figure S4. The effect of C10orf99 knockdown on the activity of the ERK1/2 signaling pathway in DLD1 cells. DLD1 cells were transfected with C10orf99 siRNA (si-C10orf99) or NC-siRNA (si-Ctrl) for 48 hours and then cells were lysed for western blot analysis. β-actin was used as an internal control. BLK, blank transfected group.

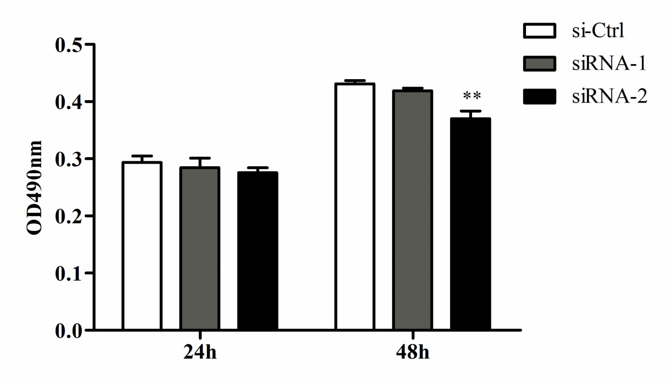

Figure S5. Effect of C10orf99 knockdown on the proliferation of HaCaT cells.
MTT assays on HaCaT cells transfected with si-Ctrl or C10orf99 siRNA. $^{**}P < 0.01$.
